# Familien im Fokus der Bundestagswahl: Ein Bekenntnis zur Systemrelevanz von Familien

**DOI:** 10.1007/s41358-021-00256-5

**Published:** 2021-04-15

**Authors:** Mathias Huebener, C. Katharina Spiess

**Affiliations:** 1 DIW Berlin, Mohrenstraße 58, 10117 Berlin (Mitte), Deutschland; 2 Freie Universität Berlin, Garystraße 21, 14195 Berlin, Deutschland

Nachdem die Familie am Anfang der Pandemie kaum eine Bedeutung hatte, wird inzwischen vielfach über die Belastung der Familien diskutiert. Fakt ist, Familien sind in der Pandemie in mehrfacher Hinsicht stark belastet. Jedoch nicht alle in gleichem Umfang. Das sollte den Parteien im Bundestagswahlkampf 2021 klar sein, wenn es darum geht die Folgen der Pandemie für Familien abzumildern und Versäumtes aufzuholen.

## Die Situation von Eltern und Kindern im Pandemiejahr

Um die Ausbreitung von Covid-19 einzudämmen, wurden im Frühjahr 2020 erstmalig Kitas und Schulen weitestgehend geschlossen. Und auch als sich im Herbst die zweite Infektionswelle in Deutschland ausbreitete, entschied die Politik zum Jahresende aufgrund der befürchteten Überlastung des Gesundheitssystems erneut Schulen und Kitas flächendeckend zu schließen. Nur im dringendsten Bedarfsfall wurde Kindern mit „systemrelevanten“ Eltern in Schulen und Kitas eine Notbetreuung angeboten. Diese drastische Maßnahme bewirkt für Familien den Zusammenbruch etablierter Familienroutinen mit weitreichenden Folgen für das Lernen und die sozialen Interaktionen, aber auch für die Möglichkeiten der Eltern, Erwerbs- und Familienarbeit zu vereinbaren. Der Großteil der Betreuung, Förderung und der Lernbegleitung von Kindern wurde an die Familien abgegeben, die neben Home-Kita und Home-Schooling vielfach auch ihrer Erwerbsarbeit aus dem Homeoffice nachgehen mussten oder erst gar kein Home-Office machen konnten und somit auch Probleme hatten Beruf und Familien zu vereinbaren. So mussten manche Eltern ihre Arbeitszeiten reduzieren, mussten teilweise ungewollt in Kurzarbeit gehen, oder verloren sogar ihren Arbeitsplatz.

Die Kita- und Schulschließungen gehören zu vielfach umstrittenen Maßnahmen der Pandemieeindämmung. Denn neben den erhofften Effekten zur Eingrenzung der Virusausbreitung und dem Schutz vor Covid-19-bedingten Gesundheitsfolgen geht diese Maßnahme mit vielerlei Nebenwirkungen für Kinder und Eltern einher. Ohne die Kita und Schule als Bildungsort sind Kinder einmal mehr auf ihr familiales Umfeld und die Unterstützung durch die Familie angewiesen. In Kombination mit der Einschränkung sozialer Kontakte und der Schließung von anderer Lern- und Begegnungsorte (wie z. B. Bibliotheken, Musikschulen, Sportvereine), veränderte die Pandemiesituation das soziale und peerbezogene Umfeld der Kinder erheblich. Dadurch sind zunächst natürlich Folgen für die kognitiven Fähigkeiten der Kinder zu erwarten, aber auch für ihre sozio-emotionale Entwicklung, ihre psychische Gesundheit und im schlimmsten Fall für ihre körperliche Unversehrtheit. Experten haben auch früh davor gewarnt, dass durch die besonders schwierige Lage für Familien auch mit einer Zunahme häuslicher Gewalt zu rechnen ist.

Pädagogische Fachkräfte, LehrerInnen, Kita-Kinder, SchülerInnen und Eltern sowie andere Akteure aus Praxis und Politik waren auf die Unterbrechung des Präsenzlernens und im Falle von Schulen des Fernunterrichts weitgehend unvorbereitet. Diese Situation hat Familien sehr viel abverlangt. Die Schließungen führten vielfach zu einem Rückgang des elterlichen Wohlbefindens, also ihrer Zufriedenheit mit dem Leben im Allgemeinen, mit dem Familienleben und mit der Kinderbetreuung (Huebener et al. 2021a; Zoch et al. 2020; Neubauer et al. 2021). Wie der Familienmonitor_Corona des DIW Berlin und Infratest dimap zeigt, gaben im Frühjahr 2021 mehr als 50 % der Eltern an, sich große Sorgen um die Bildung und die wirtschaftliche Zukunft ihrer Kinder zu machen (Huebener et al. 2021b).

## Die Pandemie droht Bildungs- und soziale Ungleichheiten zu verstärken

Jedoch sind nicht alle Kinder und Familien in gleicher Weise in dieser Situation betroffen. Sorgen um die Bildung und Zukunft der Kinder, aber auch um die eigene wirtschaftliche Situation, sind stärker ausgeprägt unter Eltern mit einem niedrigeren Bildungsabschluss. Sie gehen mit deutlichen Reduktionen des Wohlbefindens und der Zufriedenheit in verschiedenen Lebensbereichen einher (Huebener et al. 2021b). Für Kinder lassen die unterschiedlichen Bedingungen zum Aufwachsen und Lernen auch darauf schließen, dass die Auswirkungen der Pandemiesituation sich erheblich unterscheiden dürften, das haben erste wissenschaftliche Untersuchungen bereits belegt. Das häusliche Lernumfeld, auf das Kinder bei Schließungen umso mehr angewiesen sind, unterschied sich schon vor der Pandemie zwischen leistungsstärkeren und leistungsschwächeren Schülern deutlich (Huebener et al. 2021c). Leistungsschwache SchülerInnen hatten zuhause z. B. seltener einen eigenen Schreibtisch, Zugang zu Internet, und aufgrund kleinerer Wohnverhältnisse auch weniger Raum zum Rückzug. Auch der Zugang zu Lernmaterial unterschied sich während der ersten Schulschließungen teils erheblich zwischen den verschiedenen Schultypen (Huebener et al. 2021c). Zum Beispiel hatten SchülerInnen am Gymnasium eher Zugang zu Videokonferenzen als SchülerInnen an anderen Sekundarschulen. Außerdem zeigt sich, dass Privatschulen Lernmaterial häufiger digital bereitstellten. Insgesamt lassen sich deutliche Unterschiede bei Lernaktivitäten während der Pandemie feststellen, etwa zwischen Kindern von Eltern mit höherem und niedrigerem Bildungsniveau, insbesondere auch zwischen leistungsstärkeren und -schwächeren Schülerinnen (Grewenig et al. 2020). Erste Befunde z. B. aus Belgien und den Niederlanden haben bereits auf Basis standardisierter Lernstandserhebungen nachgewiesen, dass SchülerInnen durch die Schulschließungen in allen getesteten Fächern signifikante und substantielle Lernverluste erfahren haben, und die Ungleichheit innerhalb von Schulen und zwischen verschiedenen Gruppen von Schülern ansteigt. Schulen mit einer stärker benachteiligten Schülerpopulation haben größere Lernverluste erfahren (Maldonado und De Witte 2020; Engzell et al. 2020). Auch die Sorgen der Eltern um die Bildung ihrer Kinder, deren wirtschaftliche Zukunft und die eigene wirtschaftliche Situation sind bei Eltern mit niedrigeren Bildungsabschlüssen stärker ausgeprägt (Huebener et al. 2021b).

Diese und vielfache andere Beobachtungen legen nahe, dass sich die Pandemie unterschiedlich und nachhaltig auf den Lernerfolg von Kindern auswirkt und bereits bestehende Bildungsungleichheiten verstärkt werden. Damit ist auch langfristig mit einer Zunahme der sozialen Ungleichheit zu rechnen. Denn zahlreiche bildungsökonomische Studien haben belegt, dass etwa Verdienstaussichten am Arbeitsmarkt und auch die Gesundheit im Lebensverlauf durch Bildung kausal beeinflusst werden, und die Effekte gar bis in die nächste Generation hineinreichen (z. B. Lochner 2011; Huebener 2018). Eine zunehmende soziale Ungleichheit hat auch weitreichende Folgen für unser gesellschaftliches Zusammenleben.

Langfristig sind Lernausfälle also aus volkswirtschaftlicher Perspektive mit sehr hohen Kosten verbunden, die ein frühzeitiges, entschlossenes Handeln gerechtfertigt hätten. Doch obwohl von Anfang an vielerlei Vorschläge zum Gegensteuern unterbreitet wurden, erschien die Politik in Bezug auf Kitas und Schulen nicht sehr innovativ. Statt massiv in das digitale Lernen und die entsprechende Fortbildung der Lehrkräfte zu investieren, bestand im vergangenen Jahr vielfach vornehmlich schlichtweg die Hoffnung, dass die Entwicklung der Neuinfektionen keine erneuten Schließungen von Kitas und Schulen verlangen würde. Mittel- bis langfristige Vorbereitungen auf das, was dann kam, blieben vielfach aus. Es wurde vieler Orts versäumt, Schulen flächendeckend mit Luftfiltern, Masken und Schnelltests auszustatten, alternative Lernorte zu schaffen und zusätzliches Personal zu mobilisieren, etwa über pensionierte LehrerInnen oder Lehramtsanwärter um hybrides Lernen auch in kleinen Gruppen zu ermöglichen. Trotz aller Lippenbekenntnisse der Politik zur „Priorität“ für diese Gruppe bei Lockdown-Öffnungen folgten vielfach nur halbherzige Taten, deren Umsetzung aufgrund vieler bürokratischen Hürden viel zu lange dauerte.

## Familien in den Fokus der Bundestagswahlen nehmen

Nun ist es müßig, Versäumnisse der Vergangenheit zu beklagen. Stattdessen sollte der Blick in die Zukunft gerichtet werden. Dabei geht von der diesjährigen Bundestagswahl eine Gelegenheit aus, ein starkes Zeichen in Richtung Kinder und Familien zu senden, das die Versäumnisse des vergangenen Jahres eingesteht und ein umfangreiches Maßnahmenpaket vorschlägt, das die kommende Generation stärkt. Die Herausforderungen sind immens, und so sollten auch die politischen Maßnahmen sein.

Es darf nicht bei den Bemühungen bleiben, die Versäumnisse und die bereits vor der Pandemie bestehenden Baustellen im Bereich der Bildungs- und Familienpolitik im Klein-Klein zu beheben. Das ist nicht nur nicht überzeugend, sondern verkennt auch die Lasten und Herausforderungen, die diese Pandemie der zukünftigen Generation auferlegt. Denn es sind die aktuell Erwerbstätigen, aber vor allem auch die Kinder, die die resultierende Schuldenlast und die Folgekosten der Pandemie zu tragen haben. Dies kann entweder über höhere Abgaben erfolgen – oder aber über ein stärkeres Wirtschaftswachstum. Und dafür braucht es einen „Pakt Zukunft“, eine Bazooka der Familien- und Bildungspolitik, oder gar einen „Marshallplan Familie“ für die Zeit nach der Pandemie, so wie es der wissenschaftliche Beirat für Familienfragen beim Bundesfamilienministerium jüngst forderte. Wie erreicht man Familien in einer Bundestagswahl im Schatten der Pandemie?

## Thema Bildung im Bundestagswahlkampf ernst nehmen

Offensichtliches Thema für die Bundestagswahl wäre vor dem Hintergrund der beschriebenen Problematik natürlich das Thema Bildung. Doch in Normalzeiten spielt dieses Thema im Bundestagswahlkampf nur eine untergeordnete Rolle, da Bildung eben Ländersache ist. Das mag für die Organisation des Schulsystems sinnvoll sein, die Erträge von Bildung für unterschiedliche Bereiche des Lebens sind aber nicht nur Ländersache, sondern kommen auch dem Bund und der Gesellschaft als Ganzes zu Gute. Das allein rechtfertigt schon ein größeres Engagement des Bundes beim Thema Bildung in Folge der Corona-Pandemie, wie man es in Ansätzen in den vergangenen Jahren beim Kita-Ausbau, dem Ganztagsschulausbau oder der Digitalisierung von Schulen beobachten konnte. Die Pandemiesituation hat vielerlei länderübergreifende Schwachstellen im Bildungsbereich offengelegt, die es gilt entschlossen anzugehen.

Großes Gestaltungspotential liegt im Bereich der frühkindlichen Bildung, die in Kindertagesbetreuung stattfindet. Kindertageseinrichtungen wurden in der Pandemie vornehmlich für ihre Betreuungsfunktion wahrgenommen. Dabei stellen sie auch zentrale Bildungseinrichtungen dar. Insbesondere Kinder aus sozio-ökonomisch benachteiligten Gruppen profitieren von einer qualitativ guten Kita-Förderung. Neben der wichtigen Sprachförderung sind Kitas auch für die Förderung im sozio-emotionalen Bereich von großer Bedeutung. Das legt einen wesentlichen Grundstein für einen erfolgreichen Schulstart und eine gelungene Bildungsbiografie, und erzielt damit auch mittel- und langfristig hohe private und gesellschaftliche Erträge. Dies belegen diverse bildungsökonomische Studien.

## Fachkräftesicherung und Digitalisierung in Kitas

Für eine gute Qualität in Kitas ist allerdings die Sicherung von Fachkräften zentral. Die Arbeitsbedingungen von Kitapersonal waren in der Pandemiesituation teils sehr schwierig, verbunden mit hohen gesundheitlichen Risiken für die Beschäftigten. Vor dem Hintergrund des ohnehin großen Personalmangels darf es nicht riskiert werden, dass Fachkräfte in andere Bereiche abwandern, wie es bereits in der Pflege zu beobachten ist. Vielmehr sollte die Politik ein deutliches Signal senden, dass den Beruf gesellschaftlich weiter aufwertet. Eine Steigerung des Gehalts von pädagogischen Fachkräften mit akademischem Abschluss auf das Niveau von GrundschullehrerInnen wäre ein wichtiges Signal. Darüber hinaus wären bundeseinheitliche Ausbildungsstandards von hoher Bedeutung. In jedem Fall muss es darum gehen, auch die Arbeitsbedingungen so zu gestalten, dass gute pädagogische Arbeit möglich ist, dazu braucht es mehr Geld für mehr Fachkräfte.

Mit dem Einbruch der Corona-Pandemie über unser Bildungssystem wurde auch einmal mehr und deutlicher als je zuvor festgestellt, wie schlecht es um die Digitalisierung in deutschen Schulen bestellt ist. In den Bemühungen, diese Defizite auszugleichen, sollten aber auch gleich die Kitas mitgedacht werden. Denn um ihre digitale Ausstattung ist es noch schlechter bestellt als um die der Schulen. In anderen Ländern ist die Digitalisierung schon voll in der frühen Bildung angekommen, doch in Deutschland wurde vor Digitalisierungs-Überlegungen im Kita-Bereich sogar gewarnt. Dem liegt wohl ein Missverständnis zugrunde. Zunächst könnten Kindertageseinrichtungen die Technik nämlich für ein digitalisiertes Kita-Management nutzen, was den organisatorischen Alltag erheblich erleichtern dürfte. Fachkräfte könnten über digitale Medien den Austausch mit Eltern suchen, ohne private Geräte nutzen zu müssen oder Datenschutzbedenken zu haben. Digitale Medien können auch dabei helfen, Dokumentationsaufgaben im Arbeitsalltag zu erleichtern. Ergänzend können mit diesen Medien sogar Entwicklungsschwierigkeiten oder besondere Begabungen von Kindern frühzeitig identifiziert werden, was schon lange von BildungsforscherInnen gefordert wird, die sich mit Förderbedarfen in der frühen Entwicklung befassen. Auch aus einer bildungsökonomischen Perspektive birgt das großes Potenzial. Denn je früher Entwicklungsdefizite, aber auch Talente erkannt werden, umso gezielter kann darauf reagiert werden. Und natürlich geht es bei der Digitalisierung auch darum, dass Kinder frühzeitig Kompetenzen im Umgang mit digitalen Medien erwerben. Dies kann altersgerecht geschehen, dazu gibt es internationale Expertise.

Im Schulbereich sollten unbedingt die Bemühungen aufrechterhalten werden, ganztägige Schulangebote quantitativ und qualitativ auszubauen. Die gewonnene Zeit durch Ganztagsangebote kann auch genutzt werden, um Kinder spezifisch zu unterstützen und Lernrückstände aufzuholen. Aber auch hier gilt, ohne eine sehr gute Qualität können ganztägige Schulangebote nicht dazu beitragen, Bildungsungleichheiten abzubauen. Hier braucht es finanzielle Ressourcen, für gut ausgebildetes pädagogische Personal und die Umsetzung guter Konzepte. Auch hier darf es nicht um Betreuung alleine gehen.

## Familien-(arbeits)zeit schafft Raum für gesellschaftlich wertvolle Sorgearbeit

Auch in Bezug auf die Familienpolitik können im Bundestagswahlkampf starke Signale zur Würdigung der Leistungen von Familien gesendet werden. Ein Kinderbonus, wie er bereits nahezu allen Familien ohne jede Bedarfsorientierung als Pauschbetrag gewährt wurde, ist kein solches Signal, da er die unterschiedlichen Situationen von Familien völlig verkennt, und damit wertvolle Mittel für eine zielgerichtete Familienpolitik vergibt – ökonomisch gesprochen sind die Mitnahmeeffekte hoch. Vielen Familien mangelte es im Jonglieren zwischen Home-Office, Home-Kita und Home-Schooling zunächst an Zeit. Ein Angebot an Familien könnte darin bestehen, die Arbeitszeit paritätisch zu reduzieren, und damit einhergehende Gehaltseinbußen anteilig auszugleichen. Diese *Familien*arbeits*zeit* kann zwar keine verlorene Zeit zurückbringen, doch aber neue Freiräume im Alltag von Familien schaffen. Eine Teilzeitbeschäftigung beider Partner könnte sogar einer Entwicklung der Pandemie entgegenwirken, in der sich in Paaren mit tendenziell traditioneller Rollenaufteilung diese noch weiter verstärkt hat (z. B. Jessen et al. 2021).

Auch denkbar wäre die Einführung einer *Familienzeit*. Im Sinne der Elternzeit könnte sie die Begleitung von Kindern und Jugendlichen, oder aber die Pflege von Angehörigen flexibel im Lebenszyklus ermöglichen, und mit einem *Familiengeld* – im Sinne des Elterngeldes – auch diese Leistung fördern. Sie wäre mit einem Kündigungsschutz und/oder einem Anspruch auf Rückkehr in Vollzeit verbunden.

Auch könnte mit einem familienfreundlicheren Arbeitsrecht die Vereinbarkeit von Beruf und Familie noch deutlich verbessert werden. Bislang liegt es im Ermessen des Arbeitgebers, Telearbeit zu ermöglichen oder diese auch zu verweigern, selbst wenn es technisch und prozessmäßig möglich wäre. Wie die Pandemie gelehrt hat, besteht aber insbesondere in der Telearbeit ein wichtiges Flexibilisierungswerkzeug in der Vereinbarkeit von Beruf und Familie, die über die Schaffung von „Flexibilisierungsrechten“ für Männer und Frauen mit Sorgeverpflichtungen allen zustehen würden, wenn es die Voraussetzungen des Arbeitsplatzes zuließen.

## Die Systemrelevanz von Familien anerkennen

Es war und ist eine gesamtgesellschaftliche Aufgabe, die gesundheitlichen Risiken von Covid-19 insbesondere von besonders gefährdeten, vornehmlich älteren Bevölkerungsgruppen abzuwenden. Die Maßnahmen, die dazu ergriffen wurden, waren teils drastisch, und stellten mit den Kita- und Schulschließungen für Familien eine große Herausforderung dar. Für Kinder und Jugendliche haben sie – so belegen die ersten Studien – auch reale Folgen für ihre Bildung und Entwicklung, und vermutlich auch langfristig am Arbeitsmarkt und in anderen Bereichen des Lebens. Umso wichtiger ist es, dass zeitnah vermehrt in Familien investiert wird – ihre Systemrelevanz hat die Pandemie einmal mehr verdeutlicht – die Schwachstellen der Bildungs- und Familienpolitik auch. Der Bundestagswahlkampf, der vor uns liegt, wird hoffentlich zeigen, wie ernst es den unterschiedlichen Parteien mit der Bildung und der Familie ist, in jedem Fall sollte über die besten Maßnahmen in beiden Politikbereichen gestritten werden.

## References

[CR1] Engzell P, Freyd A, Verhagen M (2020). Learning inequality during the COVID-19 pandemic.

[CR2] Grewenig E, Lergetporer P, Werner K, Woessmann L, Zierow L (2020). COVID-19 and educational inequality: how school closures affect low- and high-achieving students.

[CR3] Huebener M (2018). The effects of education on health: An intergenerational perspective.

[CR5] Huebener, M., N.A. Siegel, C.K. Spieß, C. Spinner, und G.G. Wagner. 2021a. Kein „Entweder-oder“: Eltern sorgen sich im Lockdown um Bildung und Gesundheit ihrer Kinder. *DIW aktuell* 59:9. https://www.diw.de/documents/publikationen/73/diw_01.c.810996.de/diw_aktuell_59.pdf

[CR6] Huebener M, Waights S, Spiess CK, Wagner GG, Siegel NA (2021). Parental well-being in times of Covid-19 in Germany. Review of Economics of the Household.

[CR4] Huebener M, Schmitz L, Spiess CK, Zinn S, Dohmen D, Hurrelmann K (2021). Familiale, individuelle und institutionelle Einflussfaktoren auf Bildungsungleichheiten. Generation Corona? Wie Kinder und Jugendliche durch die Pandemie benachteiligt werden.

[CR7] Jessen, J., C.K. Spiess, und K. Wrohlich. 2021. Sorgearbeit während der Corona-Pandemie: Mütter übernehmen größeren Anteil – vor allem bei schon zuvor ungleicher Aufteilung. *DIW Wochenbericht* 9/2021:132–139. https://www.diw.de/documents/publikationen/73/diw_01.c.812216.de/21-9-1.pdf

[CR8] Lochner LJ, Hanushek EA, Machin S, Woessmann L (2011). Non-production benefits of education: crime, health, and good citizenship. Handbook of the Economics of Education.

[CR9] Maldonado JE, De Witte K (2020). The effect of school closures on standardized student test outcomes.

[CR10] Neubauer, A.B., A. Schmidt, A.C. Kramer, und F. Schmiedek. 2021. A little autonomy support goes a long way: Daily autonomy supportive parenting, child well-being, parental need fulfillment, and change in child, family, and parent adjustment across the adaptation to the COVID-19 pandemic. *Child Development*. 10.1111/cdev.13515 10.1111/cdev.13515 PMC8013550 33462836

[CR11] Zoch, G., A.-C. Bächmann, und B. Vicari. 2020. Who Cares When Care Closes? Care-arrangements and Parental Working Conditions during the COVID-19 Pandemic in Germany. *European Societies* 23:S576–S588. 10.1080/14616696.2020.1832700

